# Percutaneous Endoscopic Colostomy: A New Technique for the Treatment of Recurrent Sigmoid Volvulus

**DOI:** 10.4103/1319-3767.61241

**Published:** 2010-04

**Authors:** Ibrahim K. Al-Alawi

**Affiliations:** Department of Surgery, St. George, Hospital, Kogarah, Sydney, Australia

**Keywords:** Sigmoid volvulus, recurrent sigmoid volvulus, PEC

## Abstract

Sigmoid volvulus is a common cause of large bowel obstruction in western countries and Africa. It accounts for 25% of the patients admitted to the hospital for large bowel obstruction. The acute management of sigmoid volvulus is sigmoidoscopic decompression. However, the recurrence rate can be as high as 60% in some series. Recurrent sigmoid volvulus in elderly patients who are not fit for definitive surgery is difficult to manage. The percutaneous endoscopic placement of two percutaneous endoscopic colostomy tube placement is a simple and relatively safe procedure. The two tubes should be left open to act as vents for the colon from over-distending. In our opinion, this aspect is key to its success as it keeps the sigmoid colon deflated until adhesions form between the colon and the abdominal wall.

Sigmoid volvulus is a common cause of large bowel obstruction in western countries and Africa. It accounts for 25% of the patients with large bowel obstruction (LBO) admitted to the hospital. The acute management of sigmoid volvulus is sigmoidoscopic decompression. However, the recurrence rate can be as high as 60% in some series.[1]

We describe a new technique called percutaneous endoscopic colostomy (PEC) with two size 20 Fr PEC tube catheters. There are two case series and three case reports describing the technique.[2–6] All describe the use of one PEC tube of a small size. The success rate was low and therefore the technique did not gain popularity.

## CASE REPORT

A 93-year-old man with a background of atrial fibrillation (AF) on Warfarin, ischemic heart disease (IHD) and asthma. Due to the associated risk from general anesthesia (GA), non-surgical intervention was sought for the patient. He had nine documented, radiologically proven, recurrent sigmoid volvulii over a six months period. Preoperative computerized tomography (CT) scan was performed to outline the relationship of the colon to the abdominal wall and ensure no intervening small bowel. An informed consent was obtained.

**Figure 1:**
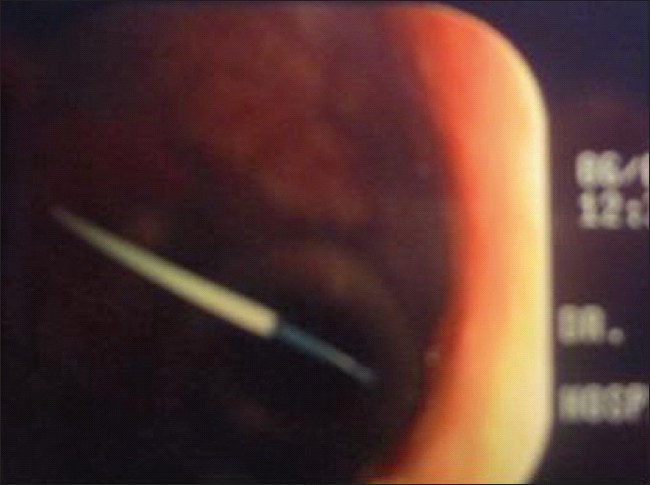
The guide wire been introduced under direct vision

**Figure 2:**
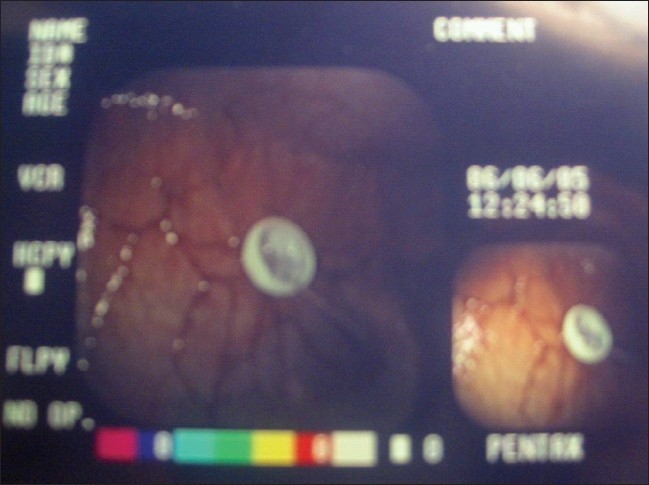
The PEC tube in place

The procedure was performed under sedation (2 mg of midazolam and 25 mcg of fentanyl) administered by anesthetists. The patient was placed in modified Lloyd Davies position. A single dose of intravenous antibiotics was given (gentamicin and metronidazole). The abdomen was prepped with aqueous Betadine. The colonoscope was introduced to the splenic flexure and then withdrawn to the level of proximal sigmoid. The lighted tip of the endoscope was seen pressing outward against the abdominal wall. Local anesthetic was infiltrated into the skin and the sheath. Using the PEG (percutaneous endoscopic gastrostomy) kit, a needle was used to enter the colon under direct vision. The wire was grasped by a snare (a polypectomy snare was used as the snare provided in the PEG tube kit was short) [Figure 1]. A 20F PEG tube was then trailed into the colon, through the abdominal wall. The position was checked with the colonoscope [Figure 2]. The same procedure was performed on the sigmoid 15 cm distally. This was slightly difficult as translumination was not obtained easily but was ultimately successful.

**Figure 3:**
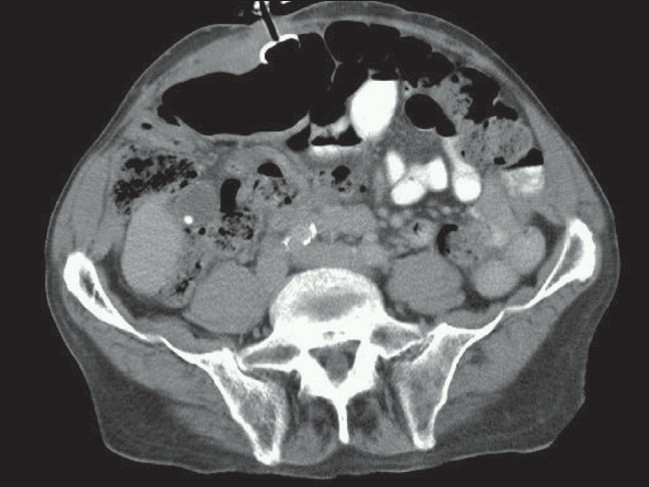
Post Op CT scan checking the position of the tubes and excluding major complications

The two tubes were left open to vent the colon and a colostomy bag was used to cover both tubes. Postoperative CT scan was done to check the position of the tubes and to ensure the absence of free gas beyond the tube insertion [Figure 3]. The patient was discharged on the second day. A year and eight months later the proximal tube eroded from the skin and was required removal in the clinic. Six months later he developed recurrence of his volvulus and a proximal tube was replaced. At two years he had no further recurrence and had a good quality of life.

## DISCUSSION

Percutaneous endoscopic colostomy (PEC) has been used in other conditions[6–8] such as acute colonic pseudo-obstruction and fecal constipation. The few contraindications to PEC are similar to PEG and include, patients who are unfit for endoscopy; inability to pass the colonoscope; failure to transilluminate, and patients with ascites owing to the concomitant potential risk of infection.[9]

Recurrent sigmoid volvulus in elderly patients who are not fit for definitive surgery is difficult to manage. The placement of two PEC tubes is a simple and relatively safe procedure. The major risk of the procedure is peritonitis, which carries almost 100% mortality since most of these patients are unfit for surgery.

Preoperative CT scan is useful to outline the anatomy of the sigmoid colon in relation to the small bowel, but is not mandatory. The two tubes should be left open to act as vents for the colon preventing overdistention. In our opinion, this is the key for success. It keeps the sigmoid colon deflated until adhesions form between the colon and the abdominal wall. The tube should be left *in situ* indefinitely.

Our case has the longest follow-up in published literature and we believe it can be offered as an alternative to open surgery in high-risk patients.
